# Treatment of unruptured cornual pregnancies by local injections of methotrexate or potassium chloride under transvaginal ultrasonographic guidance

**DOI:** 10.12669/pjms.344.14600

**Published:** 2018

**Authors:** Gorkem Tuncay, Abdullah Karaer, Ebru Inci Coskun, Rauf Melekoglu

**Affiliations:** 1 Dr. Gorkem Tuncay MD. Assistant Professor, Division of Reproductive Endocrinology, Department of Obstetrics and Gynecology, Inonu University Faculty of Medicine, Malatya, Turkey; 2 Dr. Abdullah Karaer MD. Associate Professor, Division of Reproductive Endocrinology, Department of Obstetrics and Gynecology, Inonu University Faculty of Medicine, Malatya, Turkey; 3 Dr. Ebru Inci Coskun MD. Assistant Professor, Department of Obstetrics and Gynecology, Inonu University Faculty of Medicine, Malatya, Turkey; 4 Dr. Rauf Melekoglu MD. Assistant Professor, Department of Obstetrics and Gynecology, Inonu University Faculty of Medicine, Malatya, Turkey

**Keywords:** Cornual pregnancy, Local treatment, Methotrexate, Potassium chloride

## Abstract

**Objective:**

To demonstrate the outcome of intralesional management and show the safety of local treatment of cornual pregnancy.

**Methods:**

Eight patients were treated with local methotrexate or potassium chloride injection. All patients underwent transvaginal ultrasound examination and were diagnosed by the criteria defined by Timor-Tritsch. In the case of fetal heart beat observation, potassium chloride was injected; and in the case of no heart beat detection, methotrexate was used. A follicle aspiration needle was inserted directly into the gestational sac under transvaginal guidance.

**Results:**

Although it has been considered to be a risk factor, none of the patients in our study had previous ectopic pregnancy, history of infertility / in vitro fertilization, or cornual pregnancy. One of the patients had a medical history of abortion. In four cases, methotrexate was injected, and three patients received potassium chloride as a local treatment. None of the patients had any complication in the peri- or postoperative period.

**Conclusion:**

Using a local approach, the treatment agent can reach the area of the cornual pregnancy in high concentrations. Based on this case series, a local approach seems to be an effective and fertility-sparing method for treating unruptured cornual pregnancies.

## INTRODUCTION

Cornual pregnancy, also known as interstitial pregnancy, is a rare form of ectopic pregnancy (2-4%) that occurs in a funnel-shaped area where the uterus connects to the fallopian tubes.^1^ For the management of cornual pregnancy, generally cornual resection is the preferred surgical treatment.^2^,^3^ Systemic Methotrexate (MTX) treatment^4^,^5^ may be preferable in women who want to preserve their fertility. However, MTX does have a relatively higher risk of maternal mortality and morbidity. Systemic MTX treatment requires a long hospital stay and carries the risk of systemic side effects. For that reason, intralesional potassium chloride (KCL) and/or local MTX could be an alternative treatment; these treatments have been discussed in some case series.^6^-^9^

With the advances in the resolution of ultrasonography and the development of data presentation, diagnosis of cornual pregnancy prior to rupture has greatly increased. Early diagnosis also provides a critical time for minimally invasive treatment. In this manuscript we aimed to evaluate the treatment of cornual pregnancy in patients admitted to our tertiary diagnostic and treatment center who received local KCL an/or MTX treatment. The aim of this study was to demonstrate the outcome of intralesional management and show the safety of local treatment.

## METHODS

All cornual pregnancies were treated with local MTX or KCL injection between December 2013 and February 2016. All patients underwent a transvaginal ultrasound examination with the 5-mHz transducer with doppler imaging. Three criteria, which were defined by Timor-Tritsch et al^10^, were considered in diagnosis as follows:

1) An empty uterine cavity.
2) A chorionic sac seen separately and greater than 1 cm from the most lateral edge of the uterine cavity.
3) A thin myometrial layer surrounding the gestational sac.

All patients and their partners were counseled about the management alternatives, and informed consent was obtained. Informed consent was also obtained from participants according to the Ethical Committee of Inonu University criteria.

Laparoscopic equipment was ready in the operating room during every procedure; however, surgery was not necessary in any of our patients. In the case of fetal heart beat observation, KCL was injected; and, in the case of no heart beat detection, local MTX was used. All KCL doses were 2 mL or less (from 1 mEq/mL KCL solution); and all MTX doses were 12.5 mg or less.

Under general anesthesia, a 17-gauge single lumen follicle aspiration needle was inserted into the lateral fornix and passed through the myometrium directly into the gestational sac under transvaginal guidance under sterile conditions.

## RESULTS

The patients in our study were from 28 to 38 years of age. Only case number five was primiparous; all other cases were primigravida or multiparous. The beta human Chorionic Gonadotropin (β-hCG) clearance interval in our patients was 29 to 61 days. Although it has been considered to be a risk factor, none of the patients had a previous ectopic pregnancy, history of infertility/in vitro fertilization, or cornual pregnancy. However, one patient had a history of abortion.

In four patients, MTX was injected; and three patients received KCL as a local treatment (Table-I). In case number 4, β-hCG was decreased until day 17 of injection, and then β-hCG levels drew a plato. Intramuscular MTX (1 mg/kg or m²) was also given to this patient by injection, and β-hCG was undetectable on day 61. None of the patients encountered intraabdominal/vaginal hemorrhage or infection as a complication in the peri- or postoperative period.

**Table-I:** The characteristics of eight patients diagnosed with cornual pregnancies and treated with local KCL or MTX.

Case no.	Age	Gravidity/parity	Gestational week (weeks/days)	β-hCG level before injection (mIU/mL)	β-hCG clearance interval (days)	Injected agent	Additional treatment
1	33	G5P0	6w4d	7253	45	MTX	-
2	35	G2P1	6w1d	6868	39	KCL	-
3	32	G2P1	5w6d	3243	32	MTX	-
4	29	G3P1	7w	9456	61	KCL	SYSTEMIC MTX
5	38	G1P0	7w2d	10648	58	KCL	-
6	31	G3P2	6w6d	8824	44	MTX	-
7	28	G1P0	6w1d	5246	29	MTX	-
8	34	G3P2	7w1d	12758	49	KCL	-

## DISCUSSION

Cornual pregnancy and interstitial pregnancy refer to almost the same issue. Cornual pregnancy means that the implantation of the sac has occurred approximately 1 to 1.5 cm² from the corner of uterus, and interstitial pregnancy is the implantation in the intramural site of a fallopian tube. The mortality rate in cornual pregnancy is 2% to 2.5%, and a history of ipsilataral salpengectomy, a previous ectopic pregnancy, and pelvic inflammatory disease are risk factors.^11^

Cornual pregnancies occur in 1% of all ectopic pregnancies^12^, and the ratio in in-vitro fertilization patients is 1/3600.^13^ This type of pregnancy resulted in catastrophic outcomes until recent years. This situation can especially cause a threat for the cornual region, which has a rich blood supply, and may also result in cornual rupture in late-diagnosed cases.^14^ For a long time, laparotomy or laparoscopic cornual resection was performed in such cases. However, in the last decade, opportunities for early diagnosis became much better, allowing a greater use of minimally invasive procedures, which also spare fertility.

In such cases, the criteria that Timor-Tritcsh el al^10^ defined are very useful for early diagnosis. The pregnancies that follow cornual pregnancies may result in uterine rupture due to myometrial thinning. In addition, the probability of uterine rupture was reported to be 10% to 15% after uterine surgery, regardless of reconstructive surgery.^11^ If reconstructive surgery is performed before pregnancy, the rupture risk decreases to 1%. That is a good pre-pregnancy policy for a consecutive pregnancy. Naturally, pre-pregnancy minimally invasive procedures could make reconstructive surgery unnecessary.

In local injections, bolus therapeutic medicine can reach the affected area in large amounts. In viable pregnancies, KCL is recommended. Further, in non-viable pregnancies, MTX is recommended. Both these methods were used in reported cases, and they were reported to be safe procedures.^15^

In the literature, different doses have been reported for MTX injection: 12.5 mg, 25 mg, and 100 mg.^16^ We used 12.5 mg in our patients, but larger series are needed to investigate the appropriate dosage for MTX. According to some investigators, the presence of a fetal heart beat is a contraindication for systemic MTX.^17^-^19^

Monteagudo and colleagues used local MTX or KCL for 18 viable ectopic (tubal, cornual, or cervical) pregnancies and reported successful outcomes in 16 cases.^9^ In this group, localization of ectopic pregnancy may have played a critical role. Most of the cases were cervical pregnancies, and only four patients were diagnosed with a cornual pregnancy. Surgical intervention for uterine rupture was performed in one of these cases.

Zuo et al used laparoscopy successfully in 17 cases in their series, and pointed out that there was no need to use laparotomy.^2^ Smorgick and co-workers emphasized that in their series of 31 cases (23 tubal, 5 cesarean scatris pregnancy, and 3 interstitial), patients were treated by combined local and systemic MTX, with success rates of 73.9 %, 100%, and 66.7%, respectively.^20^

Kato et al reported a case in which abdominal pain occurred after a local MTX injection of 25mg and resulted in transcervical aspiration of the gestational sac.^7^ Kato also stated that transcervical aspiration can be used in combination with laparoscopy.

Benifla and colleagues successfully treated 13 cases by local MTX and 2 cases by surgery in a 15-patient series. Furthermore, Benifla observed by hysterosalpingography a tubal patency on the ectopic side in 12 cure-obtained patients after treatment.^6^ Lin et al reported that, in unruptured cornual pregnancies, local MTX injection is a better choice than systemic MTX.^21^

Oyawoye noted that low-dose local MTX is more effective in the faster control of trophoblast growth, and is a more cost-effective method than surgery.^22^ Cerveira and co-workers commented that MTX treatment is an effective and safe method when dosage, week of pregnancy, fertility desire, and hemodynamic stability are taken into consideration.^23^ Tamarit et al reported three successful uterine artery embolizations in addition to local MTX injection.^24^

In our patients, local treatment was applied under transvaginal ultrasonographic guidance. According to the fetal viability, either KCL or MTX was provided, and the treatment was completed without any complications. In only one case was systemic MTX necessary as an additional management method. Considering these series, early diagnosed unruptured cornual pregnancies can be treated by local injections that are effective, minimally invasive, and fertility sparing as well. Larger series are needed to fully investigate the local treatment dosage, indications, and variations.

### Authors Contribution

**GT:** Conceived, designed and did statistical analysis & editing of manuscript.

**GT, RM, AK, EIC:** Did data collection and manuscript writing.

**GT:** Did review and final approval of manuscript.

## References

[1] Berek JS, Adashi EY, Hillard PA (1996). Novak's Gynecology.

[2] Zuo X, Shen A, Chen M (2012). Successful management of unruptured interstitial pregnancy in 17 consecutive cases by using laparoscopic surgery. Aus New Zea J Obstet Gynaecol.

[3] Eom JM, Choi JS, Ko JH, Lee JH, Park SH, Hong JH (2013). Surgical and obstetric outcomes of laparoscopic management for women with heterotopic pregnancy. J Obstet Gynaecol Res.

[4] Erdem M, Erdem A, Arslan M, Oc A, Biberoglu K, Gursoy R (2004). Single-dose methotrexate for the treatment of unruptured ectopic pregnancy. Arch Gynecol Obstet.

[5] Turan V, Selvi GD, Demirtas O, Akercan F, Karadadas N (2010). Kornual Ektopik Gebeligin Fetrosit ve Metotreksat ile Tedavisi. J Turk Soc Obstet Gynecol.

[6] Benifla J.L, Fernandez E, Sebban E, Darai R, Frydman P. Madelenat (1996). Alternative to surgery of treatment of unruptured interstitial pregnancy:15 cases of medical treatment. Eur J Obstet Gyneco Repro Bio.

[7] Kato S, Tanaka T, Terai Y, Yamashita Y, Ohmichi M (2011). Interstitial pregnancy treated by transcervical aspiration of the gestational sac combined with systemic and local administration of methotrexat. J Obstet Gynaecol Res.

[8] Batioglu S, Haberal A, Yeşilyurt H, Ekici E (1997). Successful treatment of cornual pregnancy by local injection of methotrexate under laparoscopic and transvaginal ultrasonographic guidance. Gynecol Obstet Invest.

[9] Monteagudo A, Minior VK, Stephenson C, Monda S, Timor-Tritsch IE (2005). Non-surgical management of live ectopic pregnancy with ultrasound-guided local injection:a case series. Ultrasound in Obstet Gyneco.

[10] Timor-Tritsch IE, Monteagudo A, Matera C, Veit CR (1992). Sonographic evolution of cornual pregnancies treated without surgery. Obstet Gynecol.

[11] Fabre-Gray A, Read M, Wardle P, James M (2014). Recurrent cornual pregnancy, successfully treated with methotrexate, following a ruptured pregnancy in the contralateral cornu. J Obstet Gynaeco.

[12] Thompson JD, Rock JA (1992). Te Linde's Operative Gynecology.

[13] Habana A, Dokras A, Giraldo JL, Jones EE (2000). Cornual heterotopic pregnancy:contemporary management options. Am J Obstet Gynecol.

[14] Damario MA, Rock JA (2003). Te-Linde's operative gynecology.

[15] Hafner T, Aslam N, Ross JA, Zosmer N, Jurkovic D (1999). The effectiveness of non-surgical management of early interstitial pregnancy:a report of ten cases and review of the literature. Ultrasound Obstet Gynecol.

[16] Ozkan ZS, Kumbak B, Sapmaz E, Simsek M (2011). Myometrial Cystic Formation after Local Methotrexate Application into Cornual Gestational Sac:A Case Report of an Unexpected Complication. Case Reports in Obstetrics and Gynecology.

[17] Glock JL, Johnson JV, Brumsted JR (1994). Efficacy and safety of single dose systemic methotrexate in the treatment of ectopic pregnancy. Fertil Steril.

[18] Henry MA, Gentry WL (1994). Single injection of methotrexate for treatment of ectopic pregnancies. Am J Obstet Gynecol.

[19] Tawfiq A, Agameya A, Claman P (2000). Predictors of treatment failure for ectopic pregnancy treated with singledose methotrexate. Fertil Steril.

[20] Smorgick N, Vaknin Z, Pansky M, Halperin R, Herman A, Maymon R (2008). Combined local and systemic methotrexate aspiration for interstitial pregnancy treatment of viable ectopic pregnancy:Outcomes of 31 cases. J Clin Ultrasound.

[21] Lin YS, Chen CL, Yuan CC, Wang PH (2007). Successful rescue of an early interstitial pregnancy after failed systemic methotrexate treatment:a case report. J Reprod Med.

[22] Oyawoye S, Chander B, Pavlovic B, Hunter J, Gadir AA (2003). Heterotopic pregnancy:successful management with aspiration of cornual/interstitial gestational sac and instillation of small dose of methotrexate. Fetal Diagn Ther.

[23] Cerveira I, Costa C, Santos F, Santos L, Cabral F (2008). Cervical ectopic pregnancy successfully treated with local methotrexate injection. Fertil Steril.

[24] Tamarit G, Lonjedo E, Gonzalez M, Tamarit S, Domingo S, Pellicer A (2010). Combined use of uterine artery embolization and local methotrexate injection in interstitial ectopic pregnancies with poor prognosis. Fertil Steril.

